# RAPD and ERIC-PCR coupled with HRM for species identification of non-dysenteriae *Shigella* species; as a potential alternative method

**DOI:** 10.1186/s13104-021-05759-6

**Published:** 2021-09-03

**Authors:** Babak Pakbin, Afshin Akhondzadeh Basti, Ali Khanjari, Leila Azimi, Wolfram Manuel Brück, Abdollah Karimi

**Affiliations:** 1 Present Address: Department of Food Hygiene and Quality of Control, Faculty of Veterinary Medicine, University of Tehran, P.O. Box 14155-6453, Tehran, Iran; 2 Pediatric Infections Research Center, Research Institute of Children’s Health, Shahid Beheshti University of Medical Sciences, Tehran, Iran; 3 Institute for Life Technologies, University of Applied Sciences Western Switzerland Valais-Wallis, 1950 Sion 2, Sierre, Switzerland

**Keywords:** PCR-HRM, Species identification, *Shigella*, Clinical samples

## Abstract

**Objective:**

Species identification of *Shigella* isolates are so prominent for epidemiological studies and infection prevention strategies. We developed and evaluated RAPD and ERIC-PCR coupled with HRM for differentiation of non-dysenteriae *Shigella* species as potential alternative methods. After isolation of eighteen *Shigella* strains from faecal specimens collected from children under 2 years of age with diarrhea (n = 143), the species of the isolates were identified by slide agglutination assay. Also, species were identified using developed RAPD-PCR-HRM and ERIC-PCR-HRM techniques. Differentiation of the data sets was measured by principal component analysis as a dimension reduction method. Then, sensitivity and specificity of the methods were evaluated.

**Results:**

We found RAPD-PCR-HRM method with high sensitivity and specificity (100 and 85% respectively) to identify non-dysenteriae *Shigella* species in clinical specimens. However, sensitivity and specificity of ERIC-PCR-HRM were evaluated 33 and 46% respectively and significantly lower than that of RAPD-PCR-HRM assay. Regardless of inherent poor reproducibility of DNA fingerprinting-based methods, RAPD-PCR-HRM assay can be considered as a potential alternative method to identify non-dysenteriae species of *Shigella* in clinical specimens. As we observed in the current study, HRM technique is more rapid, inexpensive, and sensitive than gel electrophoresis method to characterize PCR amplicons.

## Introduction

*Shigella* is a facultative anaerobe, gram-negative bacilli belonging to *Enterobacteriaceae* family and associated with intestinal and extraintestinal pathogenicity in human (Shigellosis). Four species have been characterized for *Shigella* including: *S. sonnei*, *S. flexneri*, *S. boydii*, and *S. dysenteriae*. *Shigella* species can cause mild to severe diarrhea as intestinal pathogens in adults and children (up to 28% fatality rate in children with severe symptoms); however, *S. dysenteriae* type 1, as an extraintestinal pathogen, may lead to hemorrhagic uremic syndrome [1] by production of Shiga-toxins [2]. There are significant differences between the severity and type of gastrointestinal disorders triggered by *Shigella* species [3]. Epidemiological studies revealed that identification of *Shigella* species plays a crucial role in providing clinical investigation and tracing of outbreaks caused by this pathogen [4]. The most outstanding challenge for identification of *Shigella* spp. is to differentiate species from each other [5]. Some of the differentiation methods consist of DNA hybridization, multiplex PCR, whole genome sequencing, immunocapture PCR and serological-based methods. Novel methods, still needed to be developed and optimized, are much affordable and user-friendly, with acceptable levels of specificity and sensitivity, than classical assays [6].

Sequence-based genotyping techniques are more expensive, time consuming, and complicated than other type [7]. Random amplification polymorphism DNA (RAPD) and enterobacterial repetitive intergenic consensus (ERIC) have been widely used as non-sequencing-based tools for genotyping of foodborne pathogens [8]. Due to the complicated profiles of RAPD and ERIC-PCR results, special statistical or mathematical procedures such as dimension reduction methods are strongly suggested to be carried out for further analysis of PCR products, genomic characterization and species identification [9].

High resolution melting curve analysis (HRMA) characterizes the amplified PCR products as an alternative assay of gel electrophoresis with higher precision and differentiation ability [10]. This property of HRMA makes it a promising potential candidate for analysis of the complicated PCR products [11]. At the present study, we developed RAPD and ERIC-PCR fingerprinting assays coupled with HRMA following analysis by principal component analysis (PCA) to identify and differentiate *Sh*. *sonnei*, *Sh*. *flexneri* and *Sh*. *boydii* strains isolated from clinical specimens.

## Main text

### Fecal samples collection and bacterial isolation

Children under 2 years of age with diarrhea (n = 143) in Qazvin, Iran, during December 2019 to February 2020 were referred to the central laboratory of Ghods Children Hospital of Qazvin for microbiological examination [12]. All specimens were initially cultured on isolation media including MacConkey agar (Promedia, Spain) and Salmonella-Shigella agar (SSA, Promedia, Spain) and incubated aerobically for 24 h at 37 °C. Presumptive colonies were selected for biochemical identification. Biochemically confirmed colonies were analyzed for species identification by slide agglutination test, as the gold standard method for differentiation and identification of *Shigella* species, using DIFCO *Shigella* species specific antisera kit (Difco Lab, Michigan, USA) according to the manufacturers` instructions [13]. We used *S. sonnei* ATCC 25931; *S. flexneri* ATCC 12022; and *S. boydii* ATCC 12030, purchased from Pasteur institute of Iran (Pasteur institute, Tehran, Iran), as the reference strains.

### Genomic DNA extraction

Bacterial isolates and the reference strains were inoculated into Luria‐Bertani broth (LB) and incubated at 37 °C overnight. After centrifugation at 6000 RPM for 10 min, bacterial pelletes were subjected to DNA extraction using Sinaclon gram-negative DNA extraction Kit (Sinaclon, Iran) according to the instructions described by the manufacturer. The quantity and quality of the extracted genome was evaluated using NanoDrop Spectrophotometer (Thermo Scientific, USA). Before performing PCR-HRM, concentration of all DNA templates was adjusted to 50 ng µL-1.

### RAPD and ERIC-PCR HRM

PCR-HRM method has been used for genotyping of different microbial isolates. As DNA fingerprinting methods such as RAPD and ERIC were used for differentiation of microbial species previously, we used these methods coupled with HRM to evaluate a more precise, specific and sensitive assay to identify the Shigella species in this study. We used the single random oligonucleotide primer UBC245 5`-CGC GTG CCA G-3` for RAPD-PCR and species identification of the isolates [14]. Each reaction tube contained 20 µL of mixture, including 10 µL of 2X HotStart EvaGreen PCR-HRM master mix (Solis BioDyne Co, Estonia), 0.5 µL of RAPD primer (20 pM), 1 µL of DNA template (50 ng), and distilled deionized water to the final reaction volume. HRM assay was performed using Rotor-Gene 6000 real-time PCR instrument (Corbett, Australia) as follows: initial denaturation at 95 °C for 5 min; 35 cycles of 95 °C for 1 min, 36 °C for 1 min, and 4 min at 72 °C. For performing ERIC-PCR, two primers have been used including ERIC1R 5`-ATG TAA GCT CCT GGG GAT TCA C-3` and ERIC2 5`-AAG TAA GTG ACT GGG GTG AGC G-3` [14]. ERIC-PCR was performed in 20 µL final reaction volume containing 10 µL of 2X HotStart EvaGreen PCR-HRM master mix (Solis BioDyne Co, Estonia), 1 µL of each primer (10 pM), 1 µL of DNA template (50 ng), and distilled deionized water to the final reaction volume. Rotor-Gene 6000 real-time PCR instrument (Corbett, Australia) also was employed for HRM assay. Thermal cycling program was: initial denaturation at 95 °C for 5 min followed by 30 cycles of denaturation at 95 °C for 1 min, annealing at 52 °C for 1 min, and extension at 65 °C for 8 min. HRM was carried out from 70 to 95 °C at the rate of 0.1 °C. Normalized and difference melting curves finally were achieved using the Rotor-Gene 6000 series software version 1.7 (Corbett, Australia) by normalization of melting curves between 80 and 95 °C.

### Data analysis

We used a dimension reduction method as a novel strategy for molecular subtyping by clustering and analysis of melting profiles which has previously been described by Reja et al. [15]. Principal component analysis (PCA) was used for clustering and differentiation of data sets by SPSS software version 23.0 (SPSS, Inc., Chicago, USA). Those principal components (PCs) with Eigen value (Ev) more than 1 were retained as the different groups of data set for classification and differentiation of HRM difference curves as previously described by Reja et al. [15]. Sensitivity and specificity of the methods have been evaluated as previously described by Lalkhen et al. (2008)[16].

## Results

**Fig. 1:**
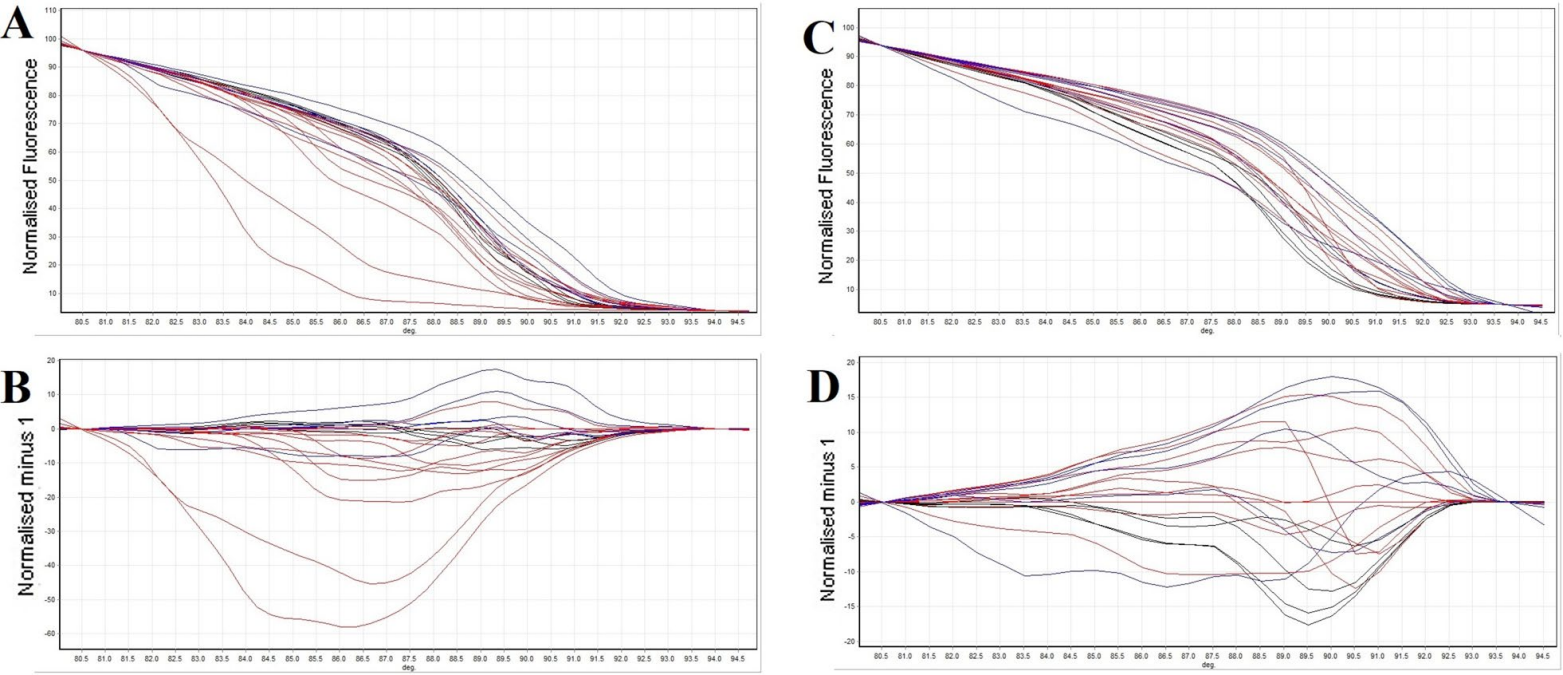
Normalized melting curves of RAPD-PCR-HRM **A** and ERIC-PCR-HRM **C** and difference melting plots of RAPD-PCR-HRM **B** and ERIC-PCR-HRM **D** for *Shigella* isolates (*S. sonnei*: red lines; *S. boydii*: black lines; *S. flexneri*: blue lines)

**Fig. 2.**
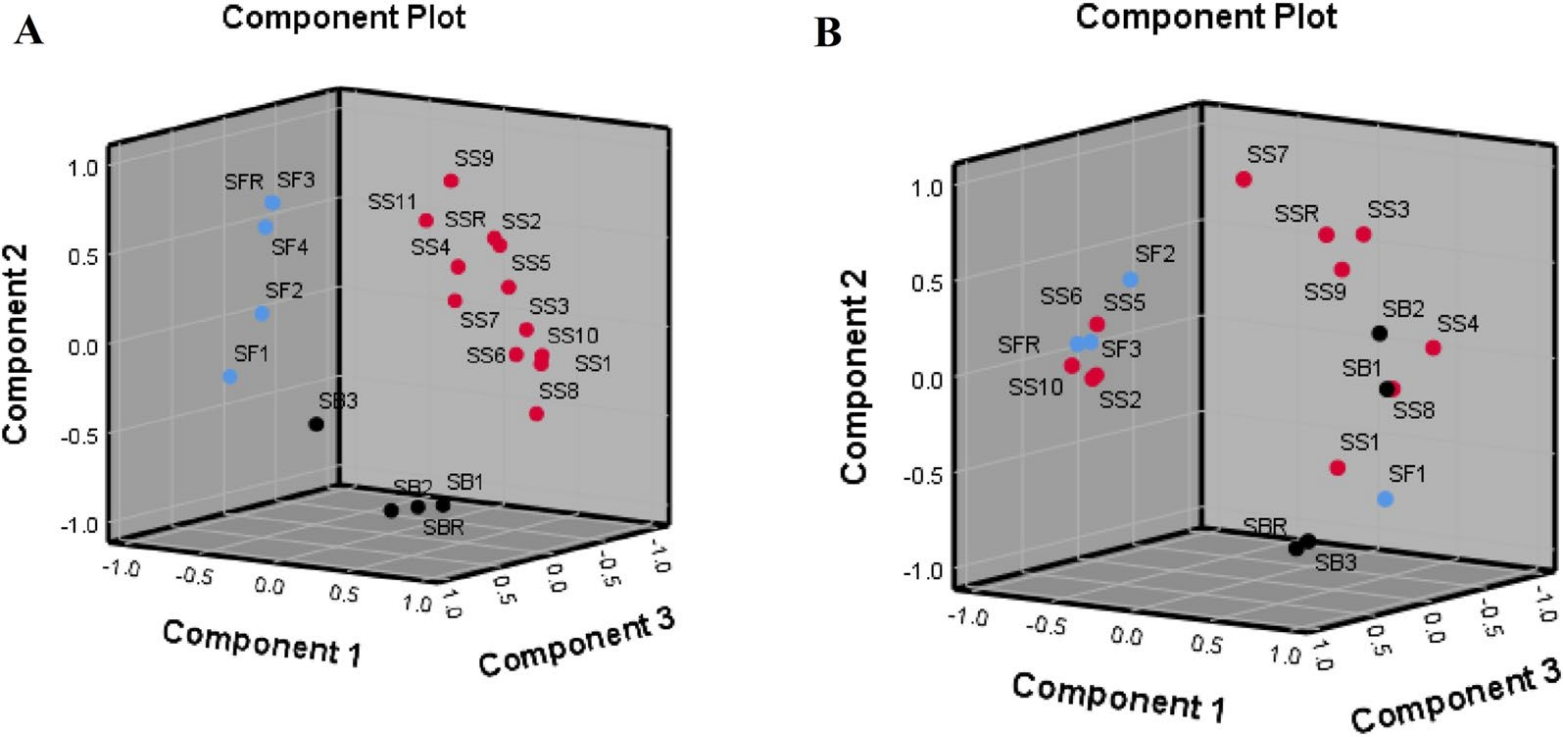
3D scores plots obtained from PCA of RAPD-PCR-HRM **A** and ERIC-PCR-HRM **B** of the *Shigella* isolates (Red spots: *S. sonnei* including SS: *S. sonnei* and SSR: *S. sonnei* reference; Black spots: *S. boydii* including SB: *S. boydii* and SBR: *S. boydii* reference; Blue spots: *S. flexneri* including SF: *S. flexneri*; SFR: *S. flexneri* reference)

We isolated and identified *Shigella* species in specimens collected from 18 out of 143 children under 2 years old with diarrhea (12.58%) consisting of 11 *S*. *sonnei* (PC3; Ev = 4.73), 3 *S*. *boydii* (PC1; Ev = 1.86), and 4 *S*. *flexneri* (PC2; Ev = 2.46) isolates using culture dependent techniques and serological tests. Then we developed and evaluated RAPD and ERIC-PCR coupled with HRM methods as a sensitive and specific potential alternative method for the species identification of *Shigella* isolates. Normalized and difference melting curves of the isolates using RAPD-PCR-HRM are shown in Fig. 1A and B, respectively. Because of extremely complicated nature of PCR products amplified in RAPD-PCR-HRM, complex melting curves were obtained and analyzed by conventional HRM software which made it difficult to differentiate melting profiles [17]. Therefore, we employed a dimension reduction method, PCA which has been recommended by many researchers, to simplify the analysis of this complex and huge data. PCA assay was performed on the data obtained from HRM difference plots. 3D scores plot obtained from PCA for the data set of difference melting curves of RAPD-PCR-HRM has been illustrated in Fig. 2A. PCA plot demonstrated three distinctive groups of curves which were considered as three species of *Shigella* among the isolates. Except one isolate which was *S*. *boydii*, species of other isolates were identified correctly using RAPD-PCR-HRM assay (11 *S*. *sonnei* (PC3; Ev = 4.73), 2 *S*. *boydii* (PC1; Ev = 1.86), and 4 *S*. *flexneri* (PC2; Ev = 2.46)).

ERIC-PCR-HRM was evaluated for species identification of *Shigella* isolates. Figure 1C and D showed normalized and difference plots of ERIC-PCR-HRM assay respectively for *Shigella* isolates. Difference plots of ERIC-PCR-HRM was analyzed using PCA assay to identify the different plot groups as three species of *Shigella* isolates. 3D scores plot obtained from PCA of the data set of ERIC-PCR-HRM has been shown in Fig. 2B. Nine species out of eighteen isolates were recognized incorrectly (6 *S*. *sonnei* (PC3; Ev = 1.66), 1 *S*. *boydii* (PC1; Ev = 3.08), and 2 *S*. *flexneri* (PC2; Ev = 1.29)). It has shown that RAPD-PCR-HRM assay is more sensitive and specific than ERIC-PCR-HRM to identify *Shigella* species when they are analyzed by PCA. Sensitivity and specificity of RAPD-PCR-HRM assay were calculated 100 and 85%, respectively. However, sensitivity and specificity of ERIC-PCR-HRM method were measured 33 and 46% respectively.

## Discussion

DNA-fingerprinting methods recently have been proposed as the method for the purpose of microbial species identification. Tulsiani et al. (2010) used HRMA of RAPD-PCR products for characterization of *Leptospira* isolates to the serovar level. They were successful to develop a new method based on RAPD-PCR-HRM assay which was an inexpensive and rapid method to identify different serotypes of *Leptospira* isolates [18]. Miller et al. (2015) developed an HRM-based assay for identification of *Pasteurellaceae* species by amplification of different regions of 16 s rDNA sequences and they found this method sensitive and specific for *Pasteurellaceae* species identification [19]. Chen et al. (2019) employed RAPD-HRM assay for differentiation of pathogenic and non-pathogenic *Escherichia coli* isolates and they found it sensitive, specific, and practical. There are limited studies about species identification and genotyping of bacterial pathogens using DNA fingerprinting methods coupled with HRM; however, several researchers applied these methods for differentiation of eukaryotic cells [17].

ERIC-PCR-HRMA has not been developed and used for molecular characterization of microbial genomes yet. We evaluated this method as a novel molecular technique for diagnostic and differentiation purpose [12]. During the HRM procedure, intercalating dyes bind to DNA and emit fluorescence because of reaction of double-strand DNA (dsDNA) and the intercalating dye. When the temperature is increased gradually, dsDNA is dissociated to single-strand DNA (ssDNA) and the decrease in fluorescence emission is measured using a highly sensitive fluorescence detector. It can be found that any difference, even a single nucleotide polymorphism (SNP), in the amplicon sequences significantly changes the melting profiles [20]. In DNA-fingerprinting methods such as RAPD and ERIC-PCR, a wide range of PCR products with different amplicon lengths are produced. These complex PCR products are considered as DNA markers to identify organisms and they can be characterized by different methods [21]. Gel electrophoresis is usually carried out for characterization of PCR products. HRM as a novel, rapid, inexpensive, sensitive and user-friendly method has recently been preferred to be used to characterize the PCR products [22].

ERIC sequences are repetitive elements located on some specific genomic regions present throughout the *Enterobacteriaceae* family genomes [23]. In RAPD-PCR, random regions of DNA are amplified during RAPD reaction and a wide range of amplicons are produced. Both used methods (RAPD and ERIC) are based on random amplification; however, higher level of variation of amplicons in RAPD-PCR should be responsible for its highly efficient differentiation capability [24]. As previously reported by several researchers, discriminatory indexes of RAPD-PCR were significantly higher than those of ERIC-PCR. It is worthy to note that the most prominent drawback of DNA-fingerprinting methods using random primers such as RAPD and ERIC-PCR is inherent poor reproducibility [25]. It is worthwhile to note that, PCR-HRM genotyping methods also can be used for differentiation of bacterial species in the future researches. However, it is greatly appreciated and suggested to improve and develop DNA-fingerprinting methods with higher reproducibility in the future studies.

## Conclusions

At the present study we introduced RAPD-PCR-HRM assay as a potential alternative method to differentiate non-dysenteriae *Shigella* species from clinical samples. We found RAPD-PCR-HRM assay more sensitive and specific than ERIC-PCR-HRM as the potential of alternative method for differentiation of non-dysenteriae *Shigella* species with sensitivity and specificity of 100 and 85%, respectively. however, future studies with more sample size and novel molecular techniques are suggested to be implemented.

## Limitations

- 18 *Shigella* isolates from clinical samples may not be sufficient to develop a species identification method.
- The most prominent drawback of RAPD and ERIC based methods is inherent poor reproducibility.
- Also, the inherent limitation of PCR-HRM assay is its inability for simultaneous identification and differentiation of microbial species in a single reaction tube.

## Data Availability

All data are available from the corresponding author on a reasonable request.

## Abbreviations

DNA: DeoxyriboNucleic Acid
PCR: Polymerase Chain Reaction
RAPD: Random Amplification of Polymorphic DNA
ERIC: Enterobacterial Repetitive Intergenic Consensus
HRMA: High Resolution Melting Curve Analysis
PCA: Principal Component Analysis

## References

[1] Murphy ER, Roßmanith J, Sieg J, Fris ME, Hussein H, Kouse AB (2020). Regulation of OmpA translation and Shigella dysenteriae virulence by an RNA thermometer. Infect Immun.

[2] Kotloff KL, Riddle MS, Platts-Mills JA, Pavlinac P, Zaidi AK (2018). Shigellosis. The Lancet.

[3] Bennish ML, Ahmed S. Shigellosis. In: Hunter's tropical medicine and emerging infectious diseases. Elsevier; 2020. p. 492–9. 10.1016/B978-0-323-55512-8.00048-X

[4] Taneja N, Mewara A (2016). Shigellosis: epidemiology in India. Indian J Med Res.

[5] Baker KS, Dallman TJ, Field N, Childs T, Mitchell H, Day M (2018). Horizontal antimicrobial resistance transfer drives epidemics of multiple Shigella species. Nat Commun.

[6] Zhou K, Kuiling S, Friedrich AW, Kooistra-Smid AM (2018). Evaluation of a culture dependent algorithm and a molecular algorithm for identification of Shigella spp., *Escherichia coli*, and entero-invasive *E. coli* (EIEC). J Clin Microbiol.

[7] Bertani G, Sardaro MLS, Neviani E, Lazzi C (2019). AFLP protocol comparison for microbial diversity fingerprinting. J Appl Genet.

[8] Shrivastava A, Singhal PK, Shrivastava P, Dash HR, Shrivastava P, Mohapatra BK (2018). Molecular diagnosis of enteric bacterial pathogens. DNA fingerprinting: advancements and future endeavors.

[9] Cui C, Li Y, Liu Y, Li X, Luo S, Zhang Z (2017). Determination of genetic diversity among Saccharina germplasm using ISSR and RAPD markers. CR Biol.

[10] Farrar JS, Wittwer C. High-resolution melting curve analysis for molecular diagnostics. In: Molecular diagnostics. Elsevier; 2017. p. 79-102. 10.1016/B978-0-12-802971-8.00006-7

[11] He P, Wang H, Luo J, Yan Y, Chen Z (2018). A real-time PCR with melting curve analysis for molecular typing of vibrio parahaemolyticus. Curr Microbiol.

[12] Codjoe FS, Brown CA, Smith TJ, Miller K, Donkor ES (2019). Genetic relatedness in carbapenem-resistant isolates from clinical specimens in Ghana using ERIC-PCR technique. PLoS ONE.

[13] Andini N, Wang B, Athamanolap P, Hardick J, Masek BJ, Thair S (2017). Microbial typing by machine learned DNA melt signatures. Sci Rep.

[14] Abolghait SK, Fathi AG, Youssef FM, Algammal AM (2020). Methicillin-resistant Staphylococcus aureus (MRSA) isolated from chicken meat and giblets often produces staphylococcal enterotoxin B (SEB) in non-refrigerated raw chicken livers. Int J Food Microbiol.

[15] Reja V, Kwok A, Stone G, Yang L, Missel A, Menzel C (2010). ScreenClust: advanced statistical software for supervised and unsupervised high resolution melting (HRM) analysis. Methods.

[16] Lalkhen AG, McCluskey A (2008). Clinical tests: sensitivity and specificity. Cont Educ Anaesthesia Crit Care Pain.

[17] Chen Y, Lai Y, Shyu D, Chang Y, Chen Z, Liao Y (2019). C-terminal part of glutamate-ammonia-ligase adenyltransferase gene identified by RAPD-HRM with 3H primer for *E. Coli* Screening. Folia Biol.

[18] Tulsiani S, Craig S, Graham G, Cobbold R, Dohnt M, Burns M-A (2010). High-resolution melt-curve analysis of random amplified polymorphic DNA (RAPD–HRM) for the characterisation of pathogenic leptospires: intra-serovar divergence, interserovar convergence, and evidence of attenuation in Leptospira reference collections. Ann Trop Med Parasitol.

[19] Miller M, Zorn J, Brielmeier M (2015). High-resolution melting curve analysis for identification of Pasteurellaceae species in experimental animal facilities. PLoS ONE.

[20] Ohshima C, Takahashi H, Iwakawa A, Kuda T, Kimura B (2017). A novel typing method for Listeria monocytogenes using high-resolution melting analysis (HRMA) of tandem repeat regions. Int J Food Microbiol.

[21] Magyar T, Gyuris É, Ujvári B, Metzner M, Wehmann E (2019). Genotyping of Riemerella anatipestifer by ERIC-PCR and correlation with serotypes. Avian Pathol.

[22] Vossen RH, White SJ, Cantsilieris S (2017). Genotyping DNA variants with high-resolution melting analysis. Genotyping.

[23] Tsai H-C, Chou M-Y, Wu C-C, Wan M-T, Kuo Y-J, Chen J-S (2018). Seasonal distribution and genotyping of antibiotic resistant strains of listeria innocua isolated from a river basin categorized by ERIC-PCR. Int J Environ Res Public Health.

[24] Yang H, Liu T, Zhang G, Chen J, Gu J, Yuan L (2017). Genotyping of Lactobacillus sanfranciscensis isolates from Chinese traditional sourdoughs by multilocus sequence typing and multiplex RAPD-PCR. Int J Food Microbiol.

[25] Staji H, Birgani SF, Raeisian B (2018). Comparative clustering and genotyping of Campylobacter jejuni strains isolated from broiler and turkey feces by using RAPD-PCR and ERIC-PCR analysis. Ann Microbiol.

